# Self‐Stabilized Hyaluronate Nanogel for Intracellular Codelivery of Doxorubicin and Cisplatin to Osteosarcoma

**DOI:** 10.1002/advs.201700821

**Published:** 2018-02-15

**Authors:** Yi Zhang, Feng Wang, Mingqiang Li, Zhiqiang Yu, Ruogu Qi, Jianxun Ding, Zhiyu Zhang, Xuesi Chen

**Affiliations:** ^1^ Department of Orthopedics The Fourth Affiliated Hospital of China Medical University Shenyang 110032 P. R. China; ^2^ Key Laboratory of Polymer Ecomaterials Changchun Institute of Applied Chemistry Chinese Academy of Sciences Changchun 130022 P. R. China; ^3^ School of Pharmaceutical Sciences Guangdong Provincial Key Laboratory of New Drug Screening Southern Medical School Guangzhou 510515 P. R. China; ^4^ State Key Laboratory of Polymer Physics and Chemistry Changchun Institute of Applied Chemistry Chinese Academy of Sciences Changchun 130022 P. R. China

**Keywords:** controlled drug release, nanogels, osteosarcoma, polysaccharides, synergistic chemotherapy

## Abstract

Osteosarcoma is one of the most serious bone malignancies with rapid speed of deterioration and low survival rate in children and teenagers. Chemotherapy is an important treatment for osteosarcoma, while the conventional small‐molecule therapeutics exhibit low efficacies and severe side effects in the clinic. Drug‐delivery platforms based on nanotechnology, particularly for self‐stabilized delivery platforms with prolonged blood circulation, enhanced intratumoral accumulation, improved antitumor efficacy, and diminished side effects, may break the deadlock on osteosarcoma chemotherapy. Here, a cisplatin (CDDP)‐crosslinked hyaluronic acid (HA) nanogel (^CDDP^HANG) is prepared for effective delivery of doxorubicin (DOX) to treat osteosarcoma. Importantly, both DOX and CDDP have led clinically used antitumor drugs, and CDDP acts as a crosslinker and ancillary anticarcinogen to prevent the premature release of DOX and to achieve synergistic therapeutic performance. Because of the enhanced stability of the nanogel, this CDDP‐crosslinked DOX‐loaded nanomedicine (^CDDP^HANG/DOX) exhibits an obviously prolonged circulation time compared to free drugs. Moreover, after valid tumor accumulation, DOX and CDDP are synergistically delivered into the tumor cells and synchronously released into the intracellular acidic environment. Based on the synergistic apoptosis‐inducing effects of DOX and CDDP, ^CDDP^HANG/DOX reveals an evidently enhanced antitumor efficacy compared to free drugs and their combination, indicating its great prospects for the chemotherapy of osteosarcoma.

## 1 Introduction

Osteosarcoma has been reported to be the sixth most common cancer in children and adolescents with just 65% of 5 year survival rate due to frequent local and distant recurrence.^1^ For a long time, chemotherapy (pre‐ and postoperative) is applied as a standard treatment procedure for osteosarcoma therapy. However, the consequent side effects existed in chemotherapy, such as hypersensitivities, gastrointestinal toxicity, and myelosuppression, which are induced by the low‐target effect and fast body metabolism of small‐molecule drug, result in poor prognosis of osteosarcoma patients.^2^ Therefore, there is an urgent need to develop a new therapeutic approach, with minimum side effects and maximum antitumor efficacy at a low dosage, for the treatment of osteosarcoma.

In the past few years, nanotechnology had witnessed a strong rise in biological, medical, and pharmaceutical applications.^3^ Specifically, the drug delivery platforms based on nanosized medicine had been extensively studied and employed for osteosarcoma treatment. These nanoparticle delivery systems possess the following unique properties: (1) excellent physicochemical properties including water‐soluble, nontoxic, and biodegradable properties; (2) prolonged blood circulation time and decreased biological clearance, inactivation, and degradation; (3) enhanced accumulation and retention in tumor tissue by enhanced permeability and retention (EPR) effect; (4) selective tumor cell targeting by receptor‐mediated targeting with a ligand.^4^

In many recently reported studies as many researches reported presently, a series of drug delivery systems based on nanotechnology exhibited their ability on improving therapeutic efficacy, while there are still some imperfect aspects limiting their further applications. Substantially, structural stability was one of the most crucial properties of nanomedicine, which helps avoid the untimely drug leakage dilemma via their delivery procedure.^5^ Poor structural stability resulted in an excessive premature drug leakage, followed by decreased therapeutic efficacy and undesirable toxicity to normal organs. Furthermore, short–long circulation time and low tumor penetration, which were crucial to the tumor inhibition efficiency, were also induced by the insufficient stability of nanomedicine. Additionally, the dose‐dependent systemic toxicity of chemotherapy drugs in nanoparticles was also an obstacle to restrict the application of nanosized drug delivery systems. The single‐drug‐loaded platforms usually need a high dose of drug to achieve the desired therapeutic effect, while the excess drug also caused the systemic side effects. Thus, it would be of significant interest to develop intelligent therapeutic nanoagents with several synergistic antitumor drugs, which are capable of possessing enhanced physiological stability and reduced side toxicity.

In this study, a biodegradable, in situ crosslinked nanogel based on hyaluronic acid (HA), a biocompatible polysaccharide, was designed to stride the multiple barriers mentioned above. Compared to other drug delivery systems, HA offers better security for in vivo study and clinical translation due to its excellent biocompatibility and biodegradability. Herein, doxorubicin (DOX) and cisplatin (CDDP), two of the most widely clinically used chemotherapy drugs with proved synergistic effects for many malignancies,^6^ were employed in this system to display their warranted antitumor effects. As previous studies reported, the antitumor effects of DOX and CDDP both relied on their interaction capability with DNA.^7^ DOX, as an anthracycline‐based topoisomerase II inhibitor, can partially hinder the efficient repair of DNA damaged by alkylating agents and has been observed to increase the efficacy of CDDP for numerous tumor cell lines.^8^

To be specific, cationic DOX was incorporated into the nanoparticle through the electronic interaction with anionic HA, obtaining HA/DOX. Meanwhile, besides the acquainted antitumor activity, CDDP was also used as a crosslinker, which chelated to the side carboxyl groups (COOH) of HA, to stabilize the drug‐loaded nanogel, avoid its premature release during circulation, prolong its circulation time, and reduce its associated side effects.^9^ Besides, we hypothesized that diminished normal organic accumulation, enhanced tumor targeting ability, and reinforced tumor penetration would be observed due to the enhanced extracellular stability. Moreover, it is expected that the intractable problem on dose‐dependent adverse effects induced by the free drugs and their combination would be solved by this co‐delivery system, due to its effectual synergistic effect and controlled intracellular drug release. The effects of crosslinking were studied by comparing the physicochemical properties, the drug release, cellular uptake, in vitro cytotoxicity, pharmacokinetics, tolerability, biodistribution, as well as the in vivo antitumor efficacy.

## 2 Results and Discussion

### 2.1 Preparation and Characterization of ^CDDP^HANG/DOX

**Scheme 1:**
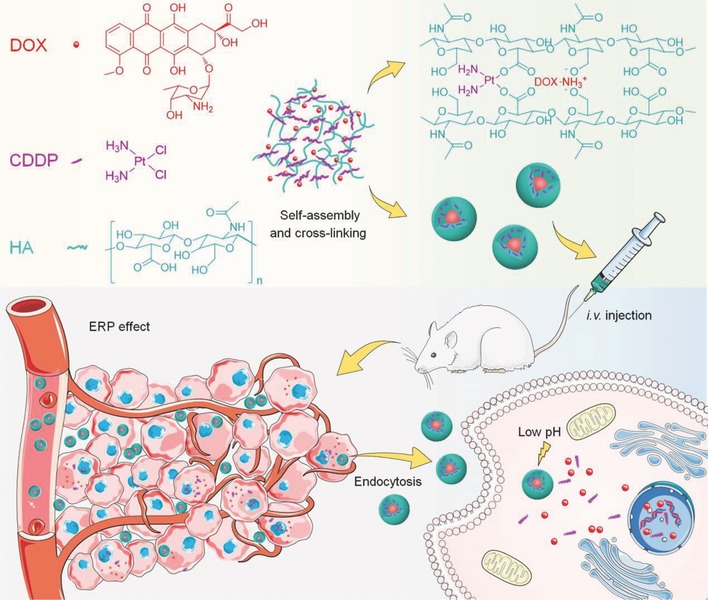
Schematic illustration for preparation, intravenous injection, in vivo circulation, selective accumulation in tumor tissue, and pH‐triggered intracellular drug release of ^CDDP^HANG/DOX.

In our study, HA, a natural polysaccharide that has been demonstrated as one of the most promising biomaterials,^10^ was utilized to encapsulate DOX and CDDP via electrostatic and chelate interactions with its side carboxyl groups, respectively (**Scheme** **1**). The number‐average molecular weight (*M*_n_) and polydispersity indices (PDI) of HA determined by gel permeation chromatography (GPC) was 16 060 g mol^−1^ and 1.7, respectively. The procedure of DOX loading was performed first by hybridizing DOX with HA in an aqueous medium with the weight ratio of 10:1. Afterward, CDDP was introduced to the solution as a crosslinker to accomplish the in situ crosslinking with three different molar ratios (10:1, 50:1, and 90:1) of COOH in HA to CDDP. These three different molar ratios were chosen based on our pilot studies. We found that further decrease of the molar ratio would induce the destabilization and precipitation of nanoparticles. On the other hand, further increase of the molar ratio would result in inadequate crosslinking, leading to the insufficient stability of the nanoparticles. Finally, the lyophilized powder of CDDP‐crosslinked DOX‐loaded nanomedicine (^CDDP^HANG/DOX) was achieved by a sequential process of dialyzing and lyophilized in darkness. It is worthy of note that all the synthetic processes were performed in an aqueous medium, thus representing a green chemistry approach.^11^

**Figure 1:**
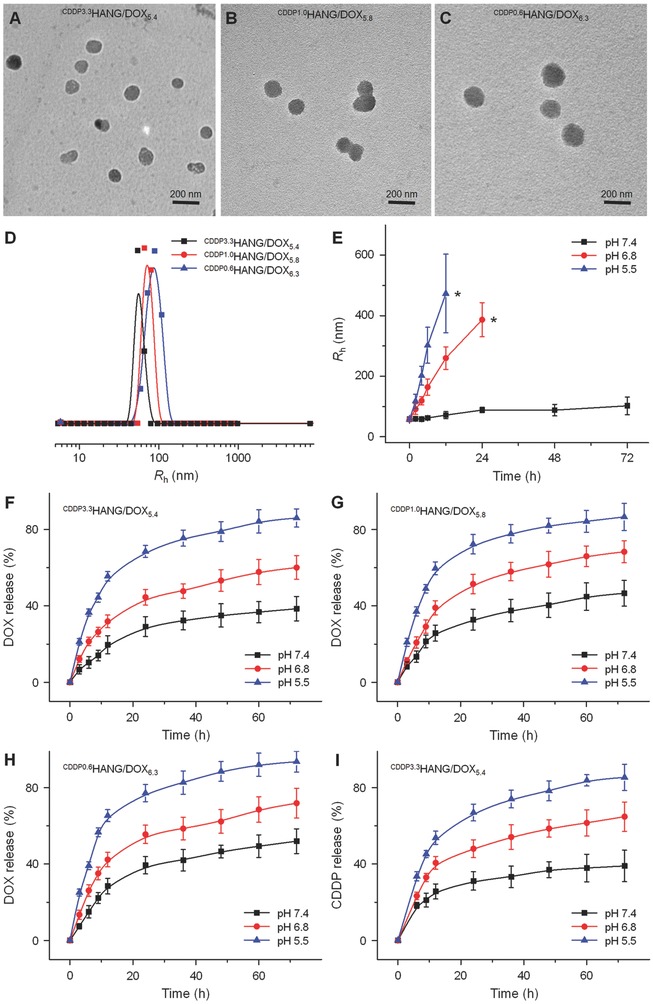
Characterization of ^CDDP^HANG/DOX. A−D) Typical TEM images and *R*_h_s of ^CDDP^HANG/DOX samples with different feeding molar ratios of [CDDP]/[COOH]. Scale bars: 200 nm. E) Size changes of ^CDDP^HANG/DOX in PBS buffer at various pH values versus time. Asterisks (*) represented the interruption of dynamic light scattering to the particles sizes. Time‐ and pH‐dependent DOX release profiles of F) ^CDDP3.3^HANG/DOX_5.4_, G) ^CDDP1.0^HANG/DOX_5.8_, H) ^CDDP0.6^HANG/DOX_6.3_, and I) the relevant CDDP release profiles of ^CDDP3.3^HANG/DOX_5.4_ in PBS at 37 °C.

After the CDDP crosslinking, our DOX‐loaded nanoparticles obtained a compacted structure. As shown in **Figure** **1**D, the hydrodynamic radius (*R*_h_) of ^CDDP^HANG/DOX decreased with the increased feeding molar ratio of CDDP from 1/90 (86.4 nm) to 1/50 (72.6 nm) and 1/10 (57.4 nm). This size shrinkage might be attributed to the enhanced crosslinking degree caused by the increased amount of CDDP. These consistent transmission electron microscopy (TEM) images also confirmed the regular changes in the particle sizes with different extents of crosslinking (Figure 1A).

Additionally, the shrinking structures also resulted in a relative decrease of the DOX's drug‐loading content (DLC) and drug‐loading efficiency (DLE) of ^CDDP^HANG/DOX, which can be explained by the steric hindrance and the depletion of the carboxylate groups of HA for drug loading. More carboxyl groups of HA would be consumed by chelation after increasing the amount of CDDP. As a result, less DOX molecules could be loaded due to the decreased carboxyl groups. As shown in **Table** **1**, for the ^CDDP^HANG/DOX with the COOH and CDDP feeding molar ratio at 1/10, its DLC and DLE were 5.4% and 51.3% for DOX, and 3.3% and 41.7% for CDDP, respectively. For the ^CDDP^HANG/DOX with the feeding ratio at 1/50, its DLC and DLE were 5.8% and 54.5% for DOX, and 1.0% and 64.3% for CDDP, respectively. For the ^CDDP^HANG/DOX with the feeding ratio at 1/90, its DLC and DLE were 6.3% and 63.2% for DOX, and 0.6% and 68.7% for CDDP, respectively. According to the different DLCs of DOX and CDDP, the above three drug‐loading nanogels were denoted as ^CDDP3.3^HANG/DOX_5.4_, ^CDDP1.0^HANG/DOX_5.8_, and ^CDDP0.6^HANG/DOX_6.3_, respectively (Table 1). For example, the DLCs of DOX and CDDP were 5.4% and 3.3%, respectively, for ^CDDP3.3^HANG/DOX_5.4_.

**Table 1:** Characterizations of DOX‐loaded nanogels with different feeding molar ratio of [CDDP]/[COOH]

Entry	Feeding molar ratio of [CDDP]/[COOH]	Resultant molar ratio of [CDDP]/[COOH]	DLC of DOX [%]	DLE of DOX [%]	DLC of CDDP [%]	DLE of CDDP [%]	*R*_h_ [nm]
^CDDP3.3^HANG/DOX_5.4_	1:10	1:20.6	5.4	51.3	3.3	41.7	57.4
^CDDP1.0^HANG/DOX_5.8_	1:50	1:69.5	5.8	54.5	1.0	64.3	72.6
^CDDP0.6^HANG/DOX_6.3_	1:90	1:115.8	6.3	63.2	0.6	68.7	86.4

### 2.2 Nanogel Stability and In Vitro DOX/CDDP Release

The stability and pH‐sensitivity of ^CDDP^HANG/DOX were verified by monitoring the changes of *R*_h_ in phosphate‐buffered saline (PBS) at different pH values. To simulate the microenvironments with various acidities in vivo, we chose pH 7.4, 6.8, and 5.5 for our study, which correspond to the pH values of the normal physiological environment, acidic tumor tissue, and intracellular endo/lysosome, respectively.^12^ ^CDDP3.3^HANG/DOX_5.4_ was taken as an example for this test. As shown in Figure 1E, ^CDDP^HANG/DOX kept a stable status without significant size change at physiological pH. However, noticeable swelling and dissociation were detected at acidic pH 6.8 and 5.5. Specifically, ^CDDP^HANG/DOX swelled from 56.9 ± 2.7 to 386.7 ± 56.3 nm within 24 h at pH 6.8, and the light‐scattering signal was not detectable owing to the particle dissociation after 36 h. Analogously, at a lower pH 5.5, the swelling and dissociation of the nanoparticle were even significant. The *R*_h_ of ^CDDP^HANG/DOX at pH 5.5 was 57.5 ± 1.3 nm at the initial state, while it rose to 473.2 ± 129.9 nm rapidly in 12 h. Due to the disassembly of ^CDDP^HANG/DOX at acidic pH, no light‐scattering signals of the particles could be detected after 12 h. The size and morphology changes of the nanogel were further confirmed by TEM (Figure S1, Supporting Information). Notably, because of the prolonged incubation time of ^CDDP^HANG/DOX in acidic environment with pH values of 6.8 or 5.5, especially for pH 5.5, its uniform spherical structure rapidly swelled and disintegrated. Finally, the nanogel disassembled and only chaotic structure was observed by TEM. Taken together, ^CDDP^HANG/DOX revealed great physiological stability, and displayed fast expansion and rupture in acidic environments corresponding to the tumor tissue and intracellular endo/lysosome.

It is crucial that nanogel can release the payload drug at the specific target site to ensure the therapeutic efficacy. The DOX release kinetics from these three samples (i.e., ^CDDP3.3^HANG/DOX_5.4_, ^CDDP1.0^HANG/DOX_5.8_, and ^CDDP0.6^HANG/DOX_6.3_) were evaluated in PBS at pH 7.4, 6.8, and 5.5 (Figure 1F–H). At the physiological pH, the initial burst release is negligible within 12 h. Moreover, the DOX release rate revealed a manifest increase as the pH decreased from 7.4 to 5.5 for ^CDDP^HANG/DOX. As a representative example, 38.5% ± 6.4% of DOX was released from the ^CDDP3.3^HANG/DOX_5.4_ after 72 h of incubation at pH 7.4, while 60.1% ± 6.2% and 86.0% ± 4.8% of DOX were released at acidic pH 6.8 and 5.5, respectively. On account of the reduced ionization degree of HA carboxyl groups under acidic conditions, the electrostatic adsorption between HA and DOX was disrupted.^13^ Thus, the drug release efficiency would have such a prominent increase under acidic conditions. Moreover, DOX had also an accelerated release at a lower pH due to its increased hydrophilicity in acid condition.^14^ This accurate and variable DOX release properties at different pH values make ^CDDP^HANG/DOX accelerate the DOX release in tumor microenvironment, and maintain a low premature leakage in physiological conditions. Therefore, ^CDDP^HANG/DOX has an optimal ability for drug delivery, which could effectively limit the organ damage and drug‐induced side effects.

Moreover, CDDP crosslinking could largely enhance the stability of the nanomedicine and might effectively prevent premature drug release following intravenous injection. The crosslinking degree of DOX‐loaded nanoparticles was correlated to the drug release kinetics. Specifically, ^CDDP3.3^HANG/DOX_5.4_, which has the highest crosslinking degree, had the least drug leakage (38.5% ± 6.4%) at pH 7.4 after 72 h compared to ^CDDP1.0^HANG/DOX_5.8_ (46.6% ± 6.7%) and ^CDDP0.6^HANG/DOX_6.3_ (52.0% ± 6.5%). Thus, as the content of CDDP increased, the DOX release rate decreased. Then, ^CDDP3.3^HANG/DOX_5.4_ was employed to illuminate pH‐dependent CDDP release profiles. As shown in Figure 1I, 39.0% ± 8.2% of CDDP was released at pH 7.4 within 72 h, while 85.4% ± 6.9% of CDDP was released at lower pH 5.5, due to the weaker chelate interaction under acidic conditions. Based on the appropriate nanoparticle size, which was optimal for EPR‐mediated passive tumor targeting,^15^ and the satisfactory specialty of drug release, which could afford an adequate amount of released drug, ^CDDP3.3^HANG/DOX_5.4_ was chosen for further studies.

### 2.3 Cell Internalization and Inhibition of Cell Proliferation

**Figure 2:**
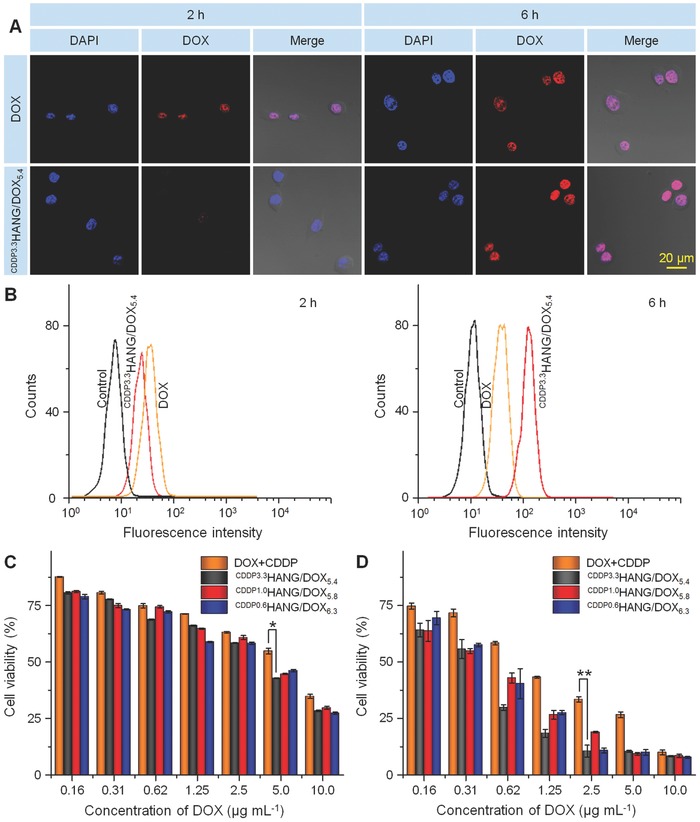
Cell uptake and cytotoxicity. A) CLSM and B) FCM analyses of K7 cells after incubation with free DOX plus CDDP and ^CDDP3.3^HANG/DOX_5.4_ for 2 or 6 h. Scale bar: 20.0 µm. C,D) In vitro cytotoxicities of free DOX plus CDDP and ^CDDP^HANG/DOX to K7 cells after incubation for C) 24 or D) 72 h. Data are presented as a mean ± standard deviation (SD; *n* = 3 for panels (C) and (D); *P < 0.05, **P < 0.01).

Confocal laser scanning microscopy (CLSM) and flow cytometry (FCM) were used to investigate the cell internalization and intracellular DOX release. As shown in **Figure** **2**A, the DOX fluorescence of ^CDDP3.3^HANG/DOX_5.4_ group was located in the nucleus, which is the same site observed for free DOX. Therefore, the drug‐encapsulated nanogel was able to release DOX intracellularly. Furthermore, after 2 or 6 h of incubation, the intracellular DOX fluorescence was observed with different extents of DOX endocytosis in mouse osteosarcoma K7 cells. Specifically, the DOX fluorescence could be apparently detected in the cells treated with free DOX after 2 h, while the fluorescent signal was actually weaker in the cells cultured with ^CDDP3.3^HANG/DOX_5.4_. However, when the culture time was prolonged to 6 h, the intracellular DOX fluorescence intensity remarkably enhanced in the ^CDDP3.3^HANG/DOX_5.4_ group, but the increase of fluorescence intensity was negligible in the cells treated with free DOX. These phenomena might be attributed to their different cell internalization mechanisms and variant fluorescence characteristics.^16^

As a small‐molecule chemotherapeutic drug, DOX could pass through the cell membrane by fast diffusion. On the contrary, ^CDDP3.3^HANG/DOX_5.4_ was taken up by the cells via endocytosis pathway, which had a temperate efficiency at the outset and then revealed the enhanced fluorescence signal after effective drug release.^17^ In addition, DOX encapsulated in the nanogel could display weaker fluorescence intensity compared with free DOX at the same concentration, as a result of self‐quenching effect of fluorescent molecule.^18^ Thus, the free DOX‐treated cells shown a stronger fluorescence intensity than that of the ^CDDP3.3^HANG/DOX_5.4_ group in the early period. However, the superiority of diffusion pathway could not sustain for a long time, and the endocytosis approach demonstrated its long‐term advantage entirely at the later period. As depicted in Figure 2B, the cell uptakes of free DOX and ^CDDP3.3^HANG/DOX_5.4_ were further confirmed by FCM, and consistent results could be obtained. In summary, ^CDDP3.3^HANG/DOX_5.4_ revealed excellent performance on K7 cell endocytosis and intracellular drug release.

The feasibility of ^CDDP^HANG/DOX in proliferation inhibition toward K7 cells in vitro was further confirmed by MTT assay. First, the cytotoxicity of HA toward K7 cells was measured (Figure S2A, Supporting Information). Only negligible cytotoxicity to the cells could be detected at all the tested concentrations of HA after 72 h of incubation, indicating its prominent biocompatibility. The in vitro cytotoxicities of free DOX plus CDDP and ^CDDP^HANG/DOX were measured after 24 and 72 h of incubation. As shown in Figure 2C, at 24 h, no significant difference of cell viabilities could be found among free DOX plus CDDP and three ^CDDP^HANG/DOX samples. However, the cytotoxicity of ^CDDP^HANG/DOX was obviously higher than that of free DOX plus CDDP at 72 h.

The half maximal inhibitory concentrations (IC_50_) directly estimated from Figure 2C,D were listed in **Table** **2**. Apparently, the IC_50_ of ^CDDP^HANG/DOX was lower than that of free DOX plus CDDP at either 24 or 72 h. To be specific, after 24 h of incubation, the IC_50_ values of ^CDDP3.3^HANG/DOX_5.4_, ^CDDP1.0^HANG/DOX_5.8_, and ^CDDP0.6^HANG/DOX_6.3_ were 1.7, 1.5, and 1.8 times lower than that of free DOX plus CDDP, respectively. Similarly, at 72 h, the IC_50_ values of ^CDDP3.3^HANG/DOX_5.4_, ^CDDP1.0^HANG/DOX_5.8_, and ^CDDP0.6^HANG/DOX_6.3_ was 2.8, 2.5, and 2.2 times lower than that of free DOX plus CDDP, respectively. These results were consistent with that demonstrated by CLSM and FCM. The enhanced cytotoxicity of ^CDDP^HANG/DOX was possibly ascribed to its enhanced stability and deferred drug release process.

**Table 2:** IC_50_ values of free DOX plus CDDP and ^CDDP^HANG/DOX at 24 and 72 h

Entry	IC_50_ [µg mL^−1^]	
	24 h	72 h
DOX plus CDDP	4.8	0.94
^CDDP3.3^HANG/DOX_5.4_	2.8	0.33
^CDDP1.0^HANG/DOX_5.8_	3.1	0.38
^CDDP0.6^HANG/DOX_6.3_	2.6	0.43

### 2.4 Pharmacokinetics and Biodistribution of ^CDDP^HANG/DOX

It is noted that nanosized micelles would be diluted once entering the blood circulation after intravenous administration. Meanwhile, the insufficient stability of drug‐loaded micelles might result in a severe drug leakage on account of its excessive dissociation.^19^ Therefore, the prolonged blood circulation and deferred blood clearance of nanomedicines were significant for efficient drug accumulation in the tumor tissue.^20^

**Figure 3:**
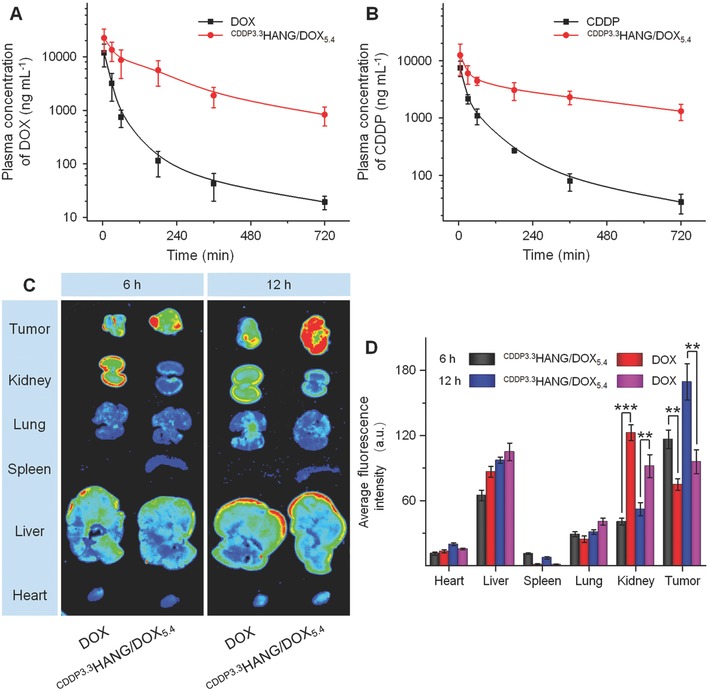
In vivo pharmacokinetics and ex vivo biodistribution studies. A,B) In vivo pharmacokinetic profiles of free DOX plus CDDP and ^CDDP3.3^HANG/DOX_5.4_ in rats. C) Ex vivo DOX fluorescence images and D) average signals of the tumors and major organs at 6 and 12 h postinjection of free DOX plus CDDP and ^CDDP3.3^HANG/DOX_5.4_ into K7 osteosarcoma‐xenografted BALB/c mice. Data are represented as mean ± SD (*n* = 3 for A, B and D, ^**^ *P* < 0.01, ^***^ *P* < 0.001).

In the present study, the pharmacokinetic profiles of free drugs and ^CDDP3.3^HANG/DOX_5.4_ were evaluated by high‐performance liquid chromatography (HPLC) and an inductively coupled plasma mass spectrometer. As shown in **Figure** **3**A,B, ^CDDP3.3^HANG/DOX_5.4_ distinctly prolonged the blood circulation of the drugs and maintained much higher plasma concentrations after the injection, as compared with the free DOX and CDDP groups (*P* < 0.05 for DOX and *P* < 0.01 for CDDP). Specifically, for the ^CDDP3.3^HANG/DOX_5.4_ group, the maximum concentrations (*C*_max_) of DOX and CDDP were 22.5 and 12.5 µg mL^−1^, respectively. However, those were 11.8 and 7.5 µg mL^−1^ in the free DOX and free CDDP groups, respectively. The area under the concentration versus time curve from 0 to final time (AUC_0–_ *_t_*) of DOX and CDDP were 60.1 and 51.4 µg (mL h)^−1^ in the ^CDDP3.3^HANG/DOX_5.4_ group, which were 5.2 and 10.1 times higher than those of the free DOX plus CDDP group, respectively. The significantly improved pharmacokinetics for ^CDDP3.3^HANG/DOX_5.4_ was probably due to their high crosslinking density that resulted in not only superior stability but also inhibited drug leakage in vivo.

Tissue biodistribution of drugs was a crucial aspect of estimating the drug effectiveness and potential organ toxicity as well as systemic side effects. Here, the ex vivo fluorescence imaging of K7 osteosarcoma tumors and main organs isolated from BALB/c mice were carried out at 6 and 12 h postinjection. All animals received care in compliance with the guidelines outlined in the Guide for the Care and Use of Laboratory Animals, and all procedures were approved by the Animal Care and Use Committee of Jilin University. As shown in Figure 3C, for free DOX‐treated mice, the fluorescence intensity in the liver and kidney was obviously stronger than that of other organs at 6 h postinjection. This can be explained by the capturing and metabolism effects by liver and kidney to the exogenous drug molecules.^21^ However, in the ^CDDP3.3^HANG/DOX_5.4_ group, the increased accumulation of DOX in other organs was detected with a conspicuous attenuation compared to free DOX, especially to the kidney, which might owe to an enhanced biocompatibility of nanogel. Moreover, due to the EPR effect of nanosized, DOX‐loaded particles, significantly stronger intratumoral DOX fluorescence could be observed in the ^CDDP3.3^HANG/DOX_5.4_ group, as compared with that of the free DOX group. The consistent results could also be obtained when the fluorescence images of the organs were compared at 12 h postinjection. Compared with the free DOX‐treated mice, an obviously enhanced DOX fluorescence intensity at tumor site was detected in the ^CDDP3.3^HANG/DOX_5.4_ ones at 12 h postinjection.

To be specific, as shown in Figure 3D, the average fluorescence intensities of organs were quantified, and the average DOX fluorescence intensity of tumor in the ^CDDP3.3^HANG/DOX_5.4_ group were 1.6 and 1.7 times higher than that in the free drug group at 6 and 12 h postinjection, respectively. These results further verified the distinct enhanced DOX accumulation in tumor site by ^CDDP3.3^HANG/DOX_5.4_ after intravenous injection, and this satisfactory biodistribution property was also helpful in reducing the systemic toxicity of the drugs.

### 2.5 In Vivo Antitumor Efficacy of ^CDDP^HANG/DOX

**Figure 4:**
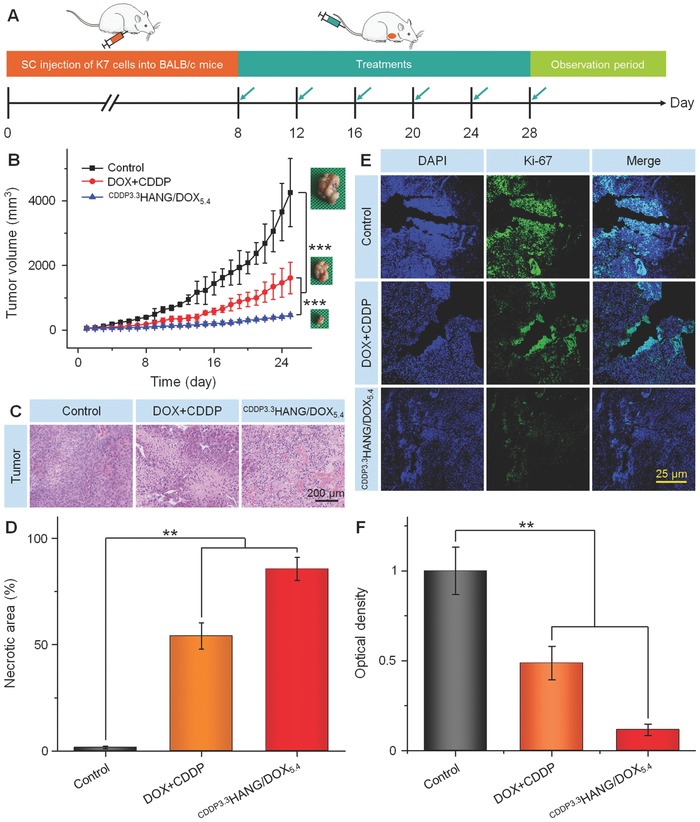
In vivo antitumor efficacies. A) Experimental schedule for tumor induction and drug treatments. B) K7 tumor growth curves, C) histopathological (i.e., H&E) analyses (magnification: 200×), and D) the necrotic areas of tumor sections from H&E of K7 osteosarcoma‐xenografted mice after treatment with PBS as the control, free DOX plus CDDP, or ^CDDP3.3^HANG/DOX_5.4_. E) Immunohistochemical (Ki‐67) analyses of tumor tissue sections after treatment of PBS as control, free DOX plus CDDP, or ^CDDP3.3^HANG/DOX_5.4_. Magnification: 100×. F) Relative optical densities of tumor sections from Ki‐67. Data are presented as a mean ± SD (*n* = 7 for panel (B), *n* = 3 for panels (D) and (F); **P < 0.01, ***P < 0.001).

The therapeutic efficacy of ^CDDP3.3^HANG/DOX_5.4_ was examined in xenografted tumor mouse model of osteosarcoma. As shown in **Figure** **4**A, when the tumor volume reached ≈50 mm^3^, free DOX plus CDDP and ^CDDP3.3^HANG/DOX_5.4_ were administered by intravenous injection (DOX dosage: 5.0 mg kg^−1^) every 4 days. As shown in Figure 4B, the tumor growth in the control group (treatment with PBS) could not be interrupted, and the volumes of these tumors were grown to around 4200 mm^3^ on average volume at a rapid speed within 25 days. In contrast, either of the other two groups treated with free DOX plus CDDP and ^CDDP3.3^HANG/DOX_5.4_ had revealed expected tumor inhibition efficiency, compared to the control group. Furthermore, ^CDDP3.3^HANG/DOX_5.4_ exhibited enhanced tumor inhibition efficiency than free DOX plus CDDP. Consistent results could be obtained through a quantitative analysis of tumor inhibition rate. After six treatments with free DOX plus CDDP and ^CDDP3.3^HANG/DOX_5.4_, a dramatic different result on tumor inhibition rate was obtained (62.1% for free DOX plus CDDP and 89.1% for ^CDDP3.3^HANG/DOX_5.4_). The tumor inhibition rate of ^CDDP3.3^HANG/DOX_5.4_ was 1.4 times higher than that of the free drug combination (free DOX plus CDDP), which can be explained by the enhanced drug accumulation retention at the tumor site, synergistic nanoparticulate drug combination, as well as the accelerated drug release at the acidic tumor microenvironment.

To further investigate the antitumor efficacy of ^CDDP3.3^HANG/DOX_5.4_, the tumors were excised from mice and sectioned for histopathological analyses after treatment. As shown in the hematoxylin and eosin (H&E) stained images (Figure 4C), various degrees of necrosis in tumor tissues were detected in the free drug combination and ^CDDP3.3^HANG/DOX_5.4_ groups, while no necrosis‐relevant indication could be found in the control group. Moreover, the semiquantitative analyses (Figure 4D) also showed that ^CDDP3.3^HANG/DOX_5.4_ induced higher tumor necrotic area (85.6% ± 5.4%) than the PBS (1.6% ± 0.7%) and free DOX plus CDDP groups (54.08% ± 6.2%), indicating its enhanced antitumor activity.

Ki‐67 was a nuclear marker for indicating cell proliferation, and also demonstrated as an optimal proliferation antigen for assessing the prognosis of cancer patients.^22^ The expression of Ki‐67 was measured in tumor sections to further evaluate the tumor apoptosis by immunohistochemistry. As shown in Figure 4E, the strongest and weakest fluorescence intensities occurred in the PBS and ^CDDP3.3^HANG/DOX_5.4_ groups, indicating the fastest and slowest cell proliferation rates of osteosarcoma, respectively. Notably, ^CDDP3.3^HANG/DOX_5.4_ induced more extensive K7 cells apoptosis in contrast to DOX plus CDDP, and the PBS treatment did not possess any significant therapeutic effect. The antiproliferation effects were further confirmed by semiquantitative evaluation of the immunohistochemical images. The fluorescence intensity of control group was set as “1.” As shown in Figure 4F, the signal of PBS group was 2.1 and 8.6 times higher than those of the free DOX plus CDDP group and ^CDDP3.3^HANG/DOX_5.4_ group, respectively. All of the above results confirmed the strong therapeutic effect of ^CDDP3.3^HANG/DOX_5.4_ in osteosarcoma.

### 2.6 In Vivo Safety of ^CDDP^HANG/DOX

Safety is another indispensable evaluation of the drug delivery systems. Whether ^CDDP3.3^HANG/DOX_5.4_ could be injected into bloodstream with negligible destruction on hemodynamics and exhibit its antitumor efficiency with reduced side effects is closely related to its further translation. In our study, we verified the safety of ^CDDP3.3^HANG/DOX_5.4_ through the hemolytic test, body weight monitoring, survival rate, organ index, and histopathological examination of several susceptible organs.

Blood compatibility was a crucial point of vesicular formulations based on whether it could be an appropriate option for intravenous injection into blood vessels.[[qv: 12a,23]] Poor blood compatibility would result in disintegration and dissolution to the membranes of red blood cells (RBCs), and this was demonstrated as a primary obstacle that restricted some kinds of drug carriers administrated through intravenous injection. In this study, hemolysis assay was employed to estimate the hemocompatibility of our nanomedicine, ^CDDP3.3^HANG/DOX_5.4_. As depicted in Figure S2B (Supporting Information), no noticeable hemolysis could be explored in all the test concentrations of ^CDDP3.3^HANG/DOX_5.4_. Such an excellent hemocompatibility makes ^CDDP3.3^HANG/DOX_5.4_ hold potential for the systemic chemotherapy after intravenous injection.

**Figure 5:**
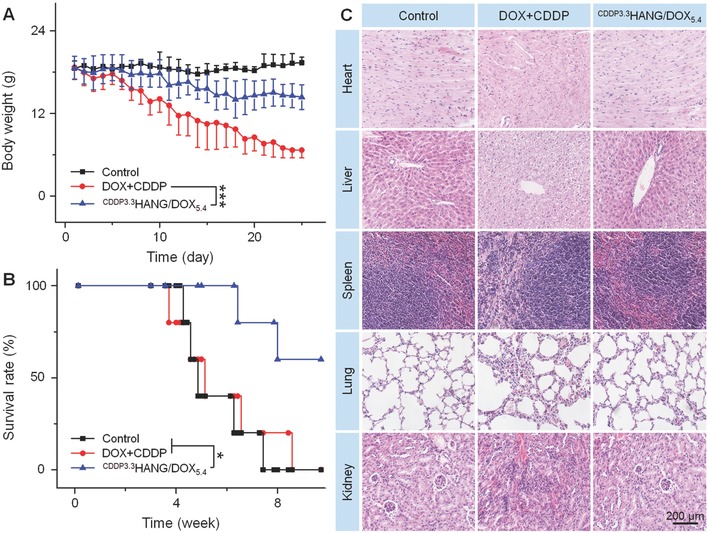
In vivo safety. A) Body weights, B) survival rates, and C) ex vivo histological analyses of main organ sections from K7 osteosarcoma‐xenografted mice after treatment with PBS as control, free DOX plus CDDP, or ^CDDP3.3^HANG/DOX_5.4_. Magnification: 200×. Scale bar: 200.0 µm. Data are presented as a mean ± SD (*n* = 7 for panel (A), *n* = 5 for panel (B); *P < 0.05, ***P < 0.001).

In our study, the body weight was monitored to estimate the potential adverse effect for all groups during the treatments. As shown in **Figure** **5**A, three groups with various treatments revealed different tendencies in body weight change. The PBS control group displayed a stable body weight, while the two other treatment groups unveiled varying degrees of body weight loss. The consequent serious side effects were induced along with the nonspecific distribution of DOX and CDDP, which further brought about a grievous body weight loss. However, thanks to the valid defending of encapsulation, ^CDDP3.3^HANG/DOX_5.4_ showed a reduced system toxicity effect, and resulted in an obviously mitigatory body weight loss.

After completion of the 25 days therapeutic efficacy study, two mice of each group were executed randomly, then the tumors and major organs were dissected and weighted. The pictures of excised organs were partially shown in Figure S3A (Supporting Information). To accurately appraise the diversity of these isolated organs, organ indices (weight ratios of organs and the whole body, mg g^−1^) were calculated. As depicted in Figure S3B (Supporting Information), a significant change was observed in the spleen. The spleen index of the free drug combination group was dramatically lower than the other two groups treated with PBS and ^CDDP3.3^HANG/DOX_5.4_, respectively, which account of the murdering of tremendous spleen cells induced by DOX.^24^ It could also further confirm that DOX encapsulated in the crosslinked conjugates had a negligible discharge, which might be attributed to the enhanced stability and tumor‐targeting releasing.

Simultaneously, the survival rate was clustered as an essential indicator to evaluate the overall therapeutic tolerance and efficacy. The residual five living mice in each treatment group were raised without any cure for this assay. Compared to PBS and free DOX plus CDDP, mice treated with ^CDDP3.3^HANG/DOX_5.4_ revealed a significantly prolonged survival term (Figure 5B; *P* < 0.05), which indicated excellent performances of ^CDDP3.3^HANG/DOX_5.4_ in abating untargeted drug accumulation and reducing systematic toxicity once again.

H&E staining of the major organs was also used to observe and estimate the long‐term toxicity of all the groups.^25^ The rationale of H&E staining was dyeing the nucleus and cytoplasm to blue/purple and red/pink by the function of hematoxylin and eosin, respectively. Through contrastive analysis of these photographs shown in Figure 5C, we found that there were no apparent morphological changes in the spleen and lung of the tumor‐bearing mice treated with free DOX plus CDDP and ^CDDP3.3^HANG/DOX_5.4_, as compared with that of the control group. However, varying degree of organ damages could be detected obviously in the heart, liver, and kidney. Especially to the heart tissue, critical texture deranging and fracture of the muscle fibers were found in the mice treated with free DOX plus CDDP. However, in the group treated with ^CDDP3.3^HANG/DOX_5.4_, valid reduced pathological changes and necrosis of heart were detected assuredly. The analogous phenomenon could also be testified in the kidney. Compare to the mice treated with ^CDDP3.3^HANG/DOX_5.4_, the mice treated with free DOX plus CDDP revealed a distinct disorder in the organizational structure of glomerulus and renal tubule. In liver, the structure of hepatocytes and hepatic lobule was necrosed and disturbed slightly for free DOX plus CDDP group, while the damage in the ^CDDP3.3^HANG/DOX_5.4_ group was more moderate. The negligible organic injury of ^CDDP3.3^HANG/DOX_5.4_ potently proved its reduced long‐term toxicity. These perfect performances showed the enormous potential of ^CDDP3.3^HANG/DOX_5.4_ for clinical application.

## 3 Conclusions

^CDDP^HANG/DOX prepared through a green and innocuous method possessed a stable structure and an outstanding ability on codelivery and subsequently controlled the release of DOX and CDDP. Accompanied with the pH‐responsive release of DOX, CDDP was also dissociative and then exhibited the synergistic antitumor effect. Moreover, after CDDP in situ crosslinking, a striking enhancement could be confirmed on its stability and tolerability. This also led to the optimized biodistribution, enhanced antitumor potency, and reduced multiorgan toxicity side effect of the drug in vivo. Based on these, ^CDDP^HANG/DOX revealed an excellent performance in either delivering drugs into tumor cells in vitro or suppressing the growth of xenografted K7 osteosarcoma in vivo. The significant tumor growth inhibition can be resulted from the prolonged blood circulation, enhanced tumor accumulation, and bioresponsive intracellular release of the drugs, after ^CDDP^HANG/DOX administrated via intravenous injection. Taken together, our CDDP crosslinked DOX‐loaded nanogel is a versatile platform for drug codelivery and holds great potential for synergistic tumor therapy.

## Conflict of Interest

The authors declare no conflict of interest.

## Supporting information

Supplementary
