# Self‐Stabilized Hyaluronate Nanogel for Intracellular Codelivery of Doxorubicin and Cisplatin to Osteosarcoma

**DOI:** 10.1002/advs.201800811

**Published:** 2018-06-19

**Authors:** Yi Zhang, Feng Wang, Mingqiang Li, Zhiqiang Yu, Ruogu Qi, Jianxun Ding, Zhiyu Zhang, Xuesi Chen

*Adv. Sci.* **2018**, *5*, 1700821

When this article was published online, the chemical structure of hyaluronate was incorrect in Scheme 1 and the position at which the amino group in doxorubicin interacts with hyaluronate was incorrectly depicted as the hydroxyl group rather than the carboxyl group. The revised scheme is shown here:


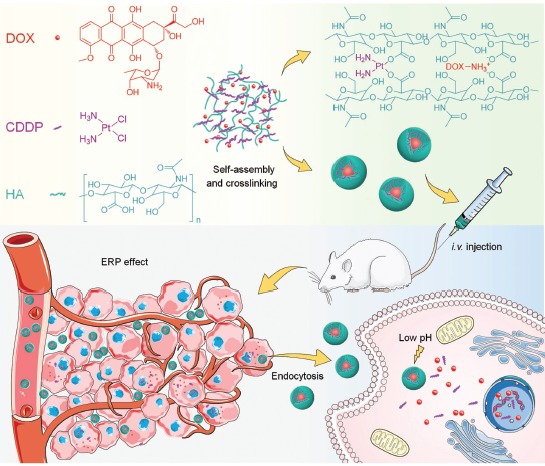


The discussions presented in the article are not affected by this error. The authors apologize for any inconvenience this may have caused.

