# Supplementary material for: Self‐Stabilized Hyaluronate Nanogel for Intracellular Codelivery of Doxorubicin and Cisplatin to Osteosarcoma
Source: Adv Sci (Weinh). 2018 Feb 15;5(5):1700821. doi: 10.1002/advs.201700821 (PMC5980114; doi:10.1002/advs.201700821)
Supplement: Supplementary file 1 — Supplementary [file ADVS-5-1700821-s001.pdf]

Copyright WILEY-VCH Verlag GmbH & Co. KGaA, 69469 Weinheim, Germany, 2018.

Supporting Information

for *Adv. Sci.,* DOI: 10.1002/advs.201700821

Self-Stabilized Hyaluronate Nanogel for Intracellular Codelivery of Doxorubicin and Cisplatin to Osteosarcoma

Yi Zhang, Feng Wang, Mingqiang Li, Zhiqiang Yu, Ruogu Qi, Jianxun Ding,* Zhiyu Zhang,* and Xuesi Chen*

Copyright WILEY-VCH Verlag GmbH & Co. KGaA, 69469 Weinheim, Germany, 2016.

Supporting Information

Self-Stabilized Hyaluronate Nanogel for Intracellular Codelivery of Doxorubicin and Cisplatin to Osteosarcoma

Yi Zhang, Feng Wang, Mingqiang Li, Zhiqiang Yu, Ruogu Qi, Jianxun Ding,* Zhiyu Zhang,* and Xuesi Chen*

Y. Zhang, Dr. F. Wang, Prof. Z. Zhang
Department of Orthopedics, the Fourth Affiliated Hospital of China Medical University, Shenyang 110032, P. R. China
E-mail: zhangzhiyudr_cmu4h@163.com

Y. Zhang, Dr. M. Li, Prof. J. Ding, Prof. X. Chen
Key Laboratory of Polymer Ecomaterials, Changchun Institute of Applied Chemistry, Chinese Academy of Sciences, Changchun 130022, P. R. China
E-mail: jxding@ciac.ac.cn

Dr. R. Qi
State Key Laboratory of Polymer Physics and Chemistry, Changchun Institute of Applied Chemistry, Chinese Academy of Sciences, Changchun 130022, P. R. China
E-mail: rqi@houstonmethodist.org

Prof. Z. Yu
School of Pharmaceutical Sciences, Guangdong Provincial Key Laboratory of New Drug Screening, Southern Medical School, Guangzhou 510515, P. R. China

Yi Zhang, Feng Wang, and Mingqiang Li contributed equally to this work.

Supplementary Materials and Methods

Materials

Low molecular weight sodium hyaluronate (Bloomage Freda Biopharm Co., Ltd., Jinan, P. R. China), doxorubicin hydrochloride (DOX·HCl, Beijing Huafeng United Technology Co., Ltd., Beijing, P. R. China), and cisplatin (Shandong Boyuan Pharmaceutical Co., Ltd, Shandong, P. R. China) were used as received. Dulbecco's modified Eagle's medium (DMEM) and fetal bovine serum (FBS) were bought from Gibco (Grand Island, NY, USA). Methyl thiazolyl tetrazolium (MTT) and 4′,6-diamidino-2-phenylindole dihydrochloride (DAPI) were sourced from Sigma-Aldrich (Shanghai, P. R. China) and used as received. Ki-67 antibodies were obtained from Abcam Company (Cambridge, UK). All the other solvents and reagents were sourced from Sigma-Aldrich (Shanghai, P. R. China) and used as received. The purified deionized water was prepared by the Milli-Q plus system (Millipore Co., Billerica, MA, USA).

Measurements

Gel permeability chromatography (GPC) analysis of hyaluronic acid (HA) was conducted on a Waters 515 system equipped with a series of liner Tskgel Super columns (AW3000 and AW5000) and a OPTILAB DSP Interferometric Refractometer (Wyatt Technology) detector (eluent: 0.1 M sodium nitrate ($NaNO_3$); flow rate: 1.0 mL min^{-1}; temperature: 35 °C). Dynamic light scattering (DLS) measurements were performed with a vertically polarized He−Ne laser (DAWN EOS, Wyatt Technology，USA). The scattering angle was fixed at 90°.

Inductively coupled plasma optical emission spectrometer (ICP-OES, iCAP 6300, Thermoscientific, USA) and inductively coupled plasma mass spectrometer (ICP-MS, Xseries II, Thermoscientific, USA) were used for quantitative determination of platinum. Transmission electron microscopy (TEM) measurements were performed on a JEOL JEM-1011 transmission electron microscope with an accelerating voltage of 100 kV. To prepare the TEM samples, the DOX conjugates was dissolved in phosphate-buffered saline (PBS) at a

concentration of 0.1 mg mL^{-1}, and then dropped on a carbon-coated copper grid and dried at room temperature for over 1 day in the air.

Preparation of ^{CDDP}HANG/DOX

^{CDDP}HANG/DOX was synthesized based on the electrostatic interaction of cationic DOX and anionic HA, and chelate interaction between the ionic HA and CDDP. To be specific, low molecular weight sodium hyaluronate (300.0 mg) lyophilized powder was dissolved in 50.0 mL of purified deionized water. And then, 5.0 mL of aqueous doxorubicin hydrochloride solution (30.0 mg) was added to the HA solution. The reaction solution was stirred (800 rpm) in the dark at room temperature for 12 h. Subsequently, predetermined CDDP was dissolved in 6.0 mL of purified deionized water and then slowly dropped into the reaction mixture mentioned above. The amount of CDDP was determined by the amount of carboxyl group of sodium hyaluronate. In this study, three molar concentration ratios (10:1, 50:1, and 90:1) of [COOH]/[CDDP] were prepared for the following studies. The reaction was performed by stirring magnetically (800 rpm) at room temperature for 72 h in darkness. The solution was dialyzed (molecular weight cut-off (MWCO) = 3500 Da) to remove the free drugs. Purified deionized water was replaced every 2 h for 24 h, and then the dialysate was lyophilized in darkness. To determine the drug loading content and loading efficiency, ^{CDDP}HANG/DOX nanoparticles were stirred in deionized water for 30 min. The drug loading content (DLC) and drug loading efficiency (DLE) of DOX and CDDP were determined by using ultraviolet-visible (UV-Vis) spectrometer and ICP-OES. DLC and DLE were calculated by **Equations S1** and **S2**, respectively.

$$\text{DLC (wt.\%)} = \frac{\text{Weight of Drug in Nanogel}}{\text{Weight of Drug-Loaded Nanogel}} \times 100\% \quad \text{(S1)}$$

$$\text{DLE (wt.\%)} = \frac{\text{Weight of Drug in Nanogel}}{\text{Total Weight of Feeding Drug}} \times 100\% \quad \text{(S2)}$$

***In Vitro* Drug Release**

To determine the release profiles of ^{CDDP}HANG/DOX in phosphate-buffered saline (PBS) at different pH values (*i.e.*, 5.5, 6.8, and 7.4), the weighed freeze-dried ^{CDDP}HANG/DOX were suspended in 10.0 mL of release media with different pH values. Then, 10.0 mL of solution was transferred into a dialysis bag (MWCO = 3500 Da). The release experiment was initiated by placing the end-sealed dialysis bag into 100.0 mL of related release medium at 37 °C with 75 rpm constant shaking. At preselected time intervals, 2.0 mL of release medium was taken out and replenished with an equal volume of fresh one. The amount of released DOX was determined using UV-Vis spectrometer at 480 nm, and the amount of released CDDP was tested by ICP-OES.

Cell Cultures, Cell Uptake, and Intracellular DOX Release

The K7 murine osteosarcoma cell line was utilized in this work and incubated in complete DMEM supplemented with 10% (*V*/*V*) FBS, penicillin (50.0 IU mL^{-1}), and streptomycin (50.0 IU mL^{-1}) at 37 °C in a 5% (*V*/*V*) carbon dioxide atmosphere.

Confocal laser scanning microscopy (CLSM) and flow cytometry (FCM) were used to analyze the cell uptake and intracellular DOX release behavior.

For CLSM, the cells were seeded on the coverslips in 6-well plates with a density of 1.5 × 10^{5} cells per well in 2.0 mL of DMEM and cultured for 12 h, and then the original medium was replaced with free DOX or ^{CDDP}HANG/DOX solution in DMEM at a final DOX concentration of 10.0 μg mL^{-1}. After incubation for 2 or 6 h, the cells were washed and fixed with 4% (*W*/*V*) PBS-buffered formaldehyde for 30 min at room temperature. And then, cell nucleus was stained with DAPI (blue) for 2 min. The cellular localization was visualized under a LSM 780 CLSM (Carl Zeiss, Jena, Germany) with 10 × eyepiece and 40 × objective.

For FCM, K7 cells with a density of 2 × 10^{5} cells per well were seeded in 6-well plates in 2.0 mL of DMEM and cultured for 12 h. Then the culture medium was replaced with free DOX or ^{CDDP}HANG/DOX in DMEM at a final DOX concentration of 10.0 μg mL^{-1}. The cells were further incubated at 37 °C for 2 or 6 h, and then washed three times with PBS. The

harvested cells were suspended in PBS and centrifuged at 1,200 rpm and 4 °C for 5 min. The supernatants were discarded, and the cells were washed again with PBS to remove the background fluorescence in the medium. After two cycles of washing and centrifugation, cells were resuspended with 500.0 μL of PBS. Flow cytometry was completed using FCM (Beckman, California, USA).

Cytotoxicity Assays

The toxicity assay of HA was implemented by a MTT assay. K7 cells were seeded in 96-well plates at a density of 8,000 cells/well and cultured for 12 h, and then the original medium was replaced with HA solution in DMEM at a final HA concentration of 0.2 mg mL^{-1}. The cells were subjected to MTT assay after being incubated for another 48 h. Briefly, 20.0 μL of MTT with a concentration of 5.0 mg mL^{-1} was added into each well and incubated for an additional 4 h. And then, the medium was replaced with 150.0 μL of dimethyl sulfoxide (DMSO). The absorbency of above solution was measured on a Bio-Rad 680 microplate reader (Hercules, CA, USA) at 490 nm. The cell viability was calculated based on **Equation S3**. The cytotoxicities of free DOX plus CDDP and ^{CDDP}HANG/DOX were evaluated by a MTT assay. K7 cells were seeded in 96-well plates at a density of 8,000 cells/well and cultured for 12 h, and then the original medium was replaced with free DOX plus CDDP or ^{CDDP}HANG/DOX solution in DMEM at a final DOX concentration of 10.0 μg mL^{-1}. The cells were subjected to MTT assay after being incubated for another 24 or 72 h.

$$\text{Cell Viability (\%)} = \frac{V_{\text{sample}}}{V_{\text{control}}} \times 100\% \qquad \text{(S3)}$$

Hemolytic Tests

The hemocompatibility levels of HA were determined according to the established standard: ISO 10993-4.[1] Briefly, the fresh rabbit blood was purchased from the Laboratory Animal Center of Jilin University. Subsequently, it was diluted in PBS, and then red blood cells (RBCs) were isolated from plasma by centrifugation at 2,500 rpm for 15 min. After the

careful wash and dilution, the suspension of RBCs at a final concentration of 2% (*V*/*V*) was added to HA solution with systematically varied concentrations, mixed by vortex, and then incubated at 37 °C in a thermostatic water bath for 3 h. PBS and Triton X-100 (1×10^4 mg mL^{-1}, a surfactant known to lyse RBCs) were used as negative and positive controls, respectively. Then, RBCs were centrifuged at 3,000 rpm for 10 min, and then 100.0 mL of the supernatant of each sample was transferred to a 96-well plate. The free hemoglobin in the supernatant was measured with a Bio-Rad 680 microplate reader at 540 nm. The hemolysis ratio of RBCs was calculated using **Equation S4**.

$$\text{Hemolytic Rate (\%)} = \frac{A_{\text{sample}} - A_{\text{negative control}}}{A_{\text{postive control}} - A_{\text{negative control}}} \times 100\% \qquad \text{(S4)}$$

In Equation S4, A_{sample}, $A_{\text{negitive control}}$, and $A_{\text{positive control}}$ were denoted as the absorbencies of sample, and negative and positive controls, respectively.

Pharmacokinetics

Sprague-Dawley rats (230 − 250 g) purchased from the Laboratory Animal Center of Jilin University were randomly divided into two groups (*n* = 3). Free DOX plus CDDP and $^{\text{CDDP3.3}}$HANG/DOX$_{5.4}$ at an equivalent DOX dose of 15.0 mg per kg body weight (mg (kg BW)$^{-1}$) in 2.0 mL of PBS was administered intravenously *via* the lateral tail vein. At defined time periods (5, 30, 60, 180, 360, and 720 min), blood samples were collected from the orbital cavity, heparinized, and centrifuged to obtain the plasma. The DOX concentrations of the above samples were detected through a high-performance liquid chromatography (HPLC) method reported previously [2]. Briefly, a 200.0 μL of plasma sample was deproteinized with 1.0 mL of methanol and 20.0 μL of daunorubicin hydrochloride at a concentration of 10.0 μg mL^{-1} as the internal standard. Then, the solution was vortexed for 10 min and subsequently centrifuged at 13,000 rpm for 10 min. After that, 500.0 μL of supernatant was collected and dried *via* a stream of nitrogen at 35 °C. The dried samples were dissolved in the mobile phase for further HPLC analyses. A fluorescence detector (Waters 2475 Multi λ Fluorescence

Detector, USA) was used in the Waters liquid chromatographic system (Waters e2695 Separations Module, USA) with the excitation and emission wavelengths set at 472 nm and 592 nm, respectively. The data was analyzed using PKSolver version 2.0 software (China Pharmaceutical University, Nanjing, P. R. China). The CDDP concentrations of the different plasma samples were obtained by detecting the concentrations of Pt. Plasma sample was heating with nitric acid to decompose the plasma samples, and the platinum contents were determined by ICP-MS.

***Ex Vivo* DOX Fluorescence Imaging**

Free DOX plus CDDP and ^{CDDP}HANG/DOX were injected into K7 osteosarcoma-xenografted BALB/c mice *via* lateral tail vein with a dose of 5.0 mg (kg BW)$^{-1}$ DOX equivalent when tumors grew to about 200 mm^3. The mice were sacrificed at 6 or 12 h post-injection. The tumors and major organs (*i.e.*, the heart, liver, spleen, lung, and kidney) were excised immediately and subsequently washed with PBS three times for *ex vivo* imaging of DOX fluorescence on a FX PRO *in vivo* Imaging System (Eastman Kodak Company, USA). In the imaging system, a light-tight box equipped with a 150 W halogen lamp and an excitation filter system (430 − 510 nm) was employed to collect DOX fluorescence. The contribution of autofluorescence was identified, separated, and removed in the analyzed images, and the average signals were also quantitatively analyzed using a Maestro™ 2.4 software.

Animal Procedures

The female BALB/C mice were obtained from the Laboratory Animal Center of Jilin University, and used at 8 weeks of age. All animals received care incompliance with the guidelines outlined in the Guide for the Care and Use of Laboratory Animals, and all procedures were approved by the Animal Care and Use Committee of Jilin University. The tumor-bearing mice were prepared through the subcutaneous injection in the armpits of right anterior limbs with 0.2 mL of cell suspension containing 2.0×10^6 mouse osteosarcoma K7 cells in PBS.

In Vivo Antitumor Efficiency

Female BALB/C mice with the average weight of 20 g were used to evaluate the *in vivo* antitumor efficacy (n = 7 for each group). The osteosarcoma carcinoma model was generated by the subcutaneous injection of K7 cells (2.5 × 10^6) into the mice with 0.1 mL PBS. When all the tumors grew up to approximately 50 mm^3, mice were randomly divided into 3 groups and then treated with free DOX plus CDDP and $^{CDDP3.3}HANG/DOX_{5.4}$ with a dose of 5.0 mg $(kg\ BW)^{-1}$ DOX equivalent, and PBS on day 1, 4, 7, 10, 14, and 17 by intravenous injection. The tumor size was measured with vernier calipers every day, and the tumor volume (V; mm^3) was calculated using **Equation S5**, and the tumor inhibition rate was calculated using **Equation S6**.

$$V\ (mm^3) = \frac{L \times S^2}{2} \qquad \text{(S5)}$$

In Equation S5, L and S (mm) were the largest and smallest diameters of tumor, respectively.

$$\text{Tumor Inhibition Rate (\%)} = \frac{V_{control} - V_{sample}}{V_{control}} \times 100\% \qquad \text{(S6)}$$

In Equation S6, V_{sample} and $V_{control}$ were denoted as the tumor volumes in sample and control groups, respectively. In addition, the body weight was monitored simultaneously as an indicator of systemic toxicity, and the survival rate was monitored from the day after treatment.

Histological and Immunohistochemical Analyses

At the 3rd day after the last injections, the mice were sacrificed, and then their tumors and major organs (*i.e.*, the heart, liver, spleen, lung, and kidney) were collected, fixed in 4% (*W*/*V*) PBS-buffered paraformaldehyde overnight, and then embedded in paraffin. The paraffin-embedded tissues were cut into ~5 mm slices for hematoxylin and eosin (H&E) staining and ~3 mm sheets for immunohistochemical analyses (*i.e.*, Ki-67 and DAPI). The histological and

immunohistochemical alterations were detected by a microscope (Nikon Eclipse Ti, Optical Apparatus Co., Ardmore, PA, USA) and subsequently analyzed with ImageJ software (National Institutes of Health, Bethesda, Maryland).

Statistical Analyses

All experiments were performed at least three times and expressed as means ± standard deviation (SD). Data were analyzed for statistical significance using SPSS 14.0 (SPSS Inc., Chicago, IL, USA). *$P < 0.05$ was considered statistically significant, and **$P < 0.01$ and ***$P < 0.001$ were considered highly significant.

References

[1] a) N. R. Kuznetsova, C. Sevrin, D. Lespineux, N. V. Bovin, E. L. Vodovozova, T. Meszaros, J. Szebeni, C. Grandfils, *J. Control. Release* **2012**, 160, 394; b) Q. L. Zhang, J. X. Ding, C. W. Lv, W. G. Xu, X. Sun, X. W. Meng, *Polymers* **2015**, 7, 2410.

[2] a) M. Li, Z. Tang, S. Lv, W. Song, H. Hong, X. Jing, Y. Zhang, X. Chen, *Biomaterials* **2014**, 35, 3851; b) M. Li, Z. Tang, Y. Zhang, S. Lv, H. Yu, D. Zhang, H. Hong, X. Chen, *J. Mater. Chem. B* **2014**, 2, 3490.

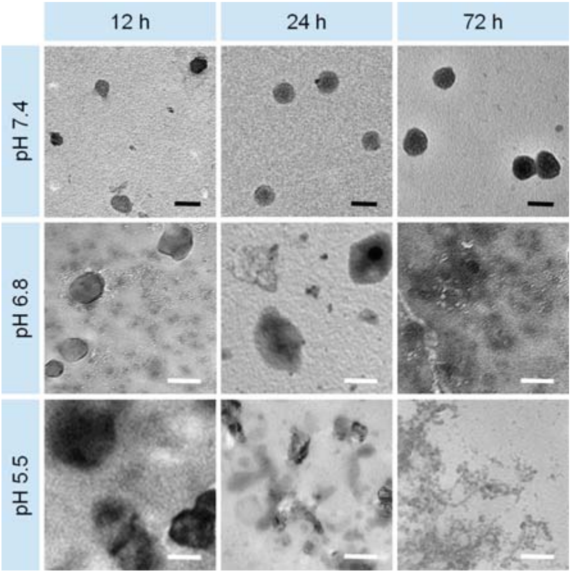

Figure S1. Size changes of ^{CDDP}HANG/DOX in PBS at various pH values *versus* time. Scale bar: black line = 200.0 nm; white line = 500.0 nm.

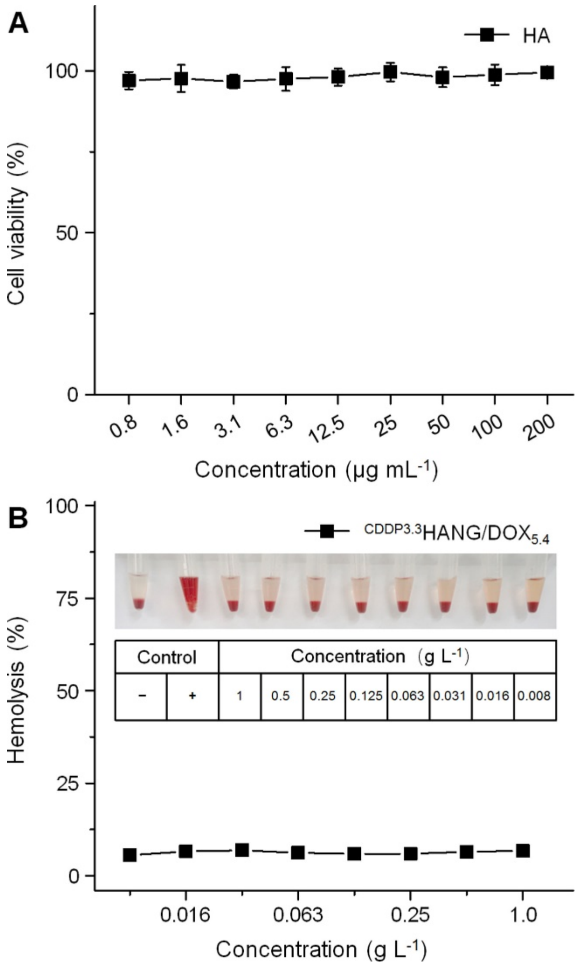

Figure S2. Biocompatibility. (A) *In vitro* cytotoxicity of HA against K7 cells after incubation for 48 h. (B) Hemolytic activity of $^{CDDP3.3}HANG/DOX_{5.4}$.

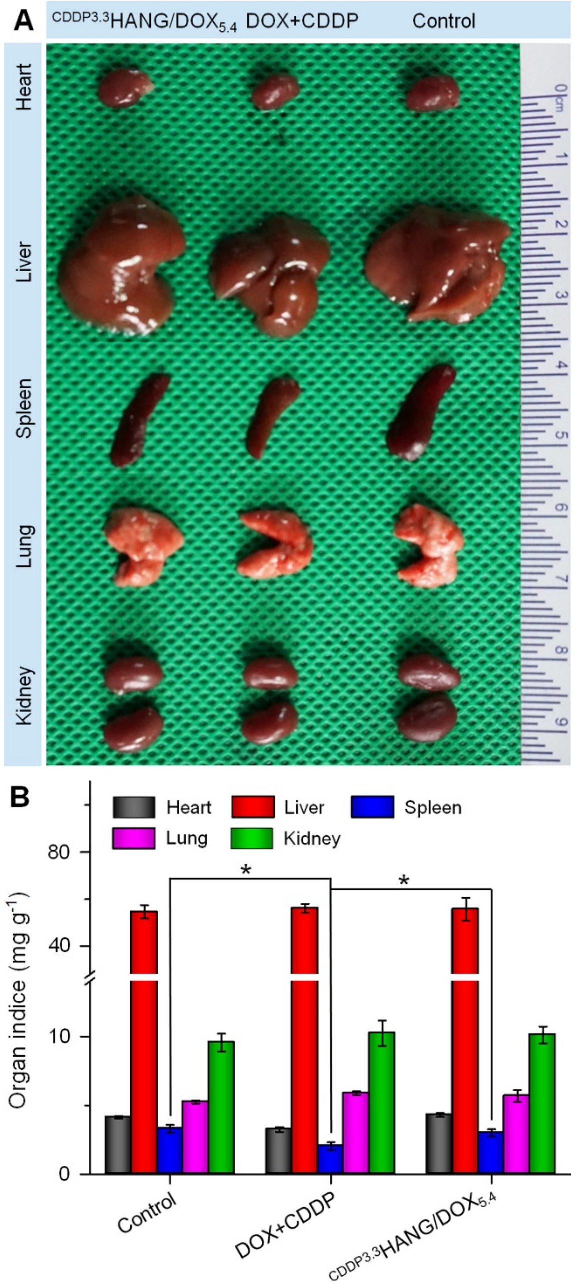

Figure S3. (A) Photos and (B) organ indices of major organs of K7 osteosarcoma-xenografted mice after treated with free DOX plus CDDP and $^{CDDP3.3}$HANG/DOX$_{5.4}$. Data are presented as a mean ± SD ($n = 3$; $^*P < 0.05$).
